# Cases of Diarrhœa, with Emaciation, Coming on after Weaning, Successfully Treated with Creosote

**Published:** 1846-12

**Authors:** 


3. *Cases of Diarrhœa, with Emaciation, coming on after weaning, successfully treated with Creosote.*—Dr. Mayes details two cases under the above head. The first, a little girl 15 months old, suffered with diarrhœa, after weaning; the following account is given of the patient's condition at the time of prescribing;

"Pale, leucophlegmatic countenance; abdomen tumid and very hot, complaining of much pain under pressure; stools excessively fœtid and dark colored, also frequent; constant harrassing dry cough; great emaciation, so much so that the integument on the extremities seemed sufficient for a second covering; no appetite at all, and some irritability of stomach; cold drinks could be retained, but every thing else was refused. This assemblage of symptoms was indicative of a fatal termination of the case, and that speedily, unless some powerful remedy could arrest the progress of the disease. *Prescription.*—℞ Creosote, 5 drops; loaf sugar, 1 drachm; gum arabic, 1 do.; water, 2 ox. Mix intimately. A tea-spoonful was administered three times daily; at the same time the tepid bath, medicated by an astringent infusion, was used two or three times daily; after a few days the cold bath was used, medicated in the same manner. In less than three days the beneficial effects of this treatment were perceptible in the improved appearance of the alvine discharges. Her amendment from this time was rapidly progressive. The last mixture made up for her was—℞ Creosote, 6 drops; loaf sugar, gum arabic, aa. 1 drachm; carbonate of iron, ½ drachm; water, 4 ounces.—Mix. The vial to be well shaken before measuring a dose. A tea-spoonful was directed three times a day. After using this mixture, no further medical treatment was thought necessary; but a nourishing diet and exercise advised."—*South. Med. and Surg. Jour.*, in *West. Lancet.*

